# Internet Health Information Seeking and the Patient-Physician Relationship: A Systematic Review

**DOI:** 10.2196/jmir.5729

**Published:** 2017-01-19

**Authors:** Sharon Swee-Lin Tan, Nadee Goonawardene

**Affiliations:** ^1^ Center for Health Informatics Department of Information Systems National University of Singapore Singapore Singapore; ^2^ SMU -TCS iCity lab Singapore Management University Singapore Singapore

**Keywords:** Internet, information seeking, physician-patient relations, health information

## Abstract

**Background:**

With online health information becoming increasingly popular among patients, concerns have been raised about the impact of patients’ Internet health information-seeking behavior on their relationship with physicians. Therefore, it is pertinent to understand the influence of online health information on the patient-physician relationship.

**Objective:**

Our objective was to systematically review existing research on patients’ Internet health information seeking and its influence on the patient-physician relationship.

**Methods:**

We systematically searched PubMed and key medical informatics, information systems, and communication science journals covering the period of 2000 to 2015. Empirical articles that were in English were included. We analyzed the content covering themes in 2 broad categories: factors affecting patients’ discussion of online findings during consultations and implications for the patient-physician relationship.

**Results:**

We identified 18 articles that met the inclusion criteria and the quality requirement for the review. The articles revealed barriers, facilitators, and demographic factors that influence patients’ disclosure of online health information during consultations and the different mechanisms patients use to reveal these findings. Our review also showed the mechanisms in which online information could influence patients’ relationship with their physicians.

**Conclusions:**

Results of this review contribute to the understanding of the patient-physician relationship of Internet-informed patients. Our main findings show that Internet health information seeking can improve the patient-physician relationship depending on whether the patient discusses the information with the physician and on their prior relationship. As patients have better access to health information through the Internet and expect to be more engaged in health decision making, traditional models of the patient-provider relationship and communication strategies must be revisited to adapt to this changing demographic.

## Introduction

As the Internet becomes a ubiquitous part of individuals’ information lives, most people have access to and are becoming comfortable with using the Internet for their information needs [1]. In health care, the rapid proliferation of health information on the Internet has resulted in more patients turning to the Internet as their first source of health information [2-4] and acquiring knowledge on their health conditions before seeking a professional diagnosis. Patients are feeling more empowered [5,6] and are more inclined toward being involved in their health and health decision making [7]. This may thus change the way in which patients interact with and participate in consultations with their physicians and how they feel about their relationship with their physicians.

Notwithstanding the potential benefits of Internet health information seeking, some concerns have been raised about the plausible negative effects of Internet health information seeking on patients. First, as online health information content can range from being peer reviewed or professionally reviewed to personal blogs, opinions, or anecdotes of other patients, information quality can vary, and patients may not possess the necessary skills to evaluate medical information and relate it to their own health circumstances [8-10]. As a consequence, online information can lead to patients’ being misinformed, lead to distress, and increase the tendency toward self-diagnosis or self-treatment [9]. Internet-informed patients may have more questions and may request additional treatments or medications during consultations [11]. Hence, online information can add a new interpretive role to physicians’ responsibilities during consultations [12,13]. Second, when patients’ online findings do not align with physicians’ diagnosis or treatments, concerns have been raised as to how a patient’s appointment satisfaction and trust in the physician would be affected [2,8,14] and how conflicts or even arguments could occur between the physician and patient [12]. This may then result in dissatisfied patients who may seek a second opinion, change the physician, change their treatment plan [15], or self-medicate using recommendations found on the Internet [16].

As patients’ Internet health information seeking becomes more pervasive, the expectations and needs of Internet-informed patients in their interactions with their physicians are expected to change. Thus, it is pertinent to have a comprehensive understanding of the influence of online health information on the patient-physician relationship. To the best of our knowledge, no review has synthesized and analyzed how patients’ Internet health information seeking affects the patient-physician relationship. The closest reviews we have found are by McMullan [17] and Wald et al [18]. McMullan [17] examined physicians’ reactions to online information and identified 3 possibilities: (1) physicians could feel threatened by the information and respond defensively by asserting their “expert opinion,” (2) physicians and patients could collaborate in obtaining and analyzing the information, and (3) physicians could guide patients to reliable health information websites. Wald et al [18] reviewed the past literature to identify the advantages and disadvantages of Internet-acquired information, and the challenges to providing guidelines to health care providers for effective interaction with Internet-informed patients.

The focus of this review was to systematically review and synthesize the existing research on Internet health information-seeking behavior and its impact on the patient-physician relationship to give implications for future research and practice. Specifically, our review sought to understand how and in what ways the Internet information search behavior of patients prior to consultations would affect doctor-patient encounters and patients’ relationships with their doctors. Our research question was “How and in what ways does patients’ Internet health information-seeking behavior influence the patient-physician relationship?”

## Methods

### Search Procedure

We systematically searched PubMed to identify articles and citations from January 1, 2000 to October 1, 2015. We also searched articles from key medical informatics journals and information systems journals (*Journal of the American Medical Informatics Association*, *International Journal of Medical Informatics*, *Journal of Medical Internet Research*, *Journal of Health Communication*, *Information Systems Research*, *Management Information Systems Quarterly*, and *Journal of the Association for Information Science and Technology*) to include additional relevant studies.

**Figure 1:**
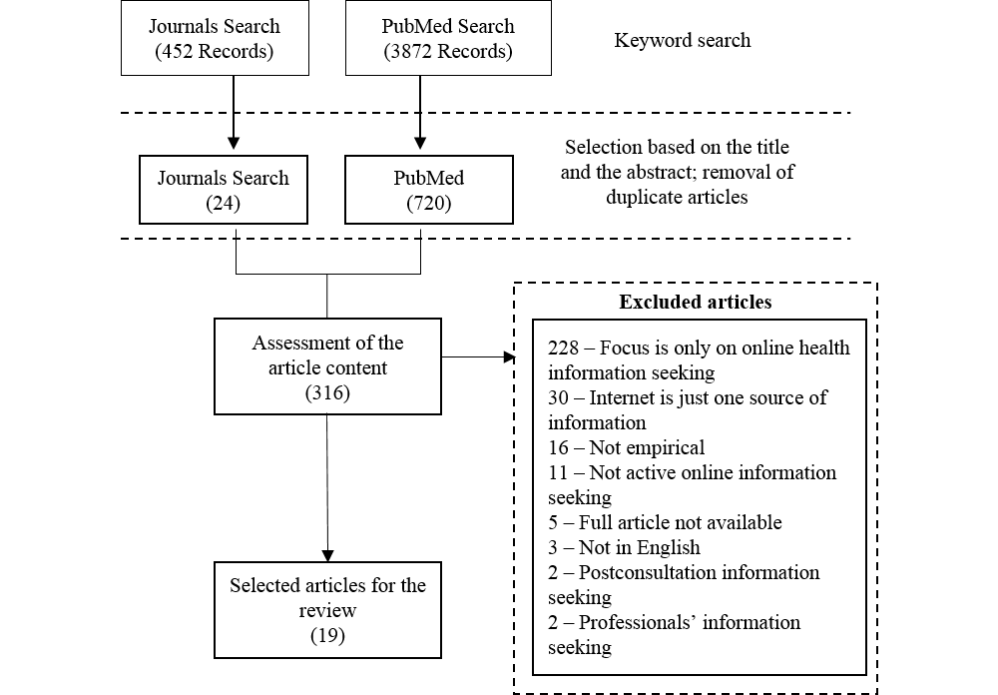
Search procedure for empirical studies on Internet-informed patients’ relationship with their physicians.

The search strategy included all possible combinations of keywords under 3 broad themes: (1) online OR Internet OR Web, (2) wellness information OR health information, (3) search* OR seek*. Further, we used the Medical Subject Headings (MeSH) “patient-physician relations” and “Internet” to perform a separate search in PubMed. PubMed searches yielded 3872 records, while journal searches yielded 452 records. We removed duplicate articles and screened the remaining articles in 2 stages. The first stage involved screening titles and abstracts to identify and exclude irrelevant articles. The remaining articles were then subjected to a second stage of screening of their main content. Figure 1 depicts the flow of the article selection procedure.

We included articles that were in English and were empirical studies focused on the Internet health information-seeking behavior of health care consumers and aspects of the patient-physician relationship. We excluded nonempirical articles, which included review articles, content assessment studies of websites, and research commentaries.

### Analysis Procedure

We conducted this systematic review to analyze published empirical studies on Internet-informed patients’ relationship with their physicians. The 19 articles selected were first assessed for research quality, and 2 researchers independently performed the quality assessment. We assessed intercoder consistency at the end. As the selected articles were mainly empirical cross-sectional studies, we used quality assessment tools that were able to assess the methodological quality, findings, and contribution of the research articles. We evaluated qualitative studies using the Critical Appraisal Skills Programme quality assessment tool [19], which consists of 10 questions that assess the quality of the research methodology and the contribution of the qualitative studies. We assessed quantitative studies using a customized coding scheme that consists of 14 questions adapted from 3 well-established quality assessment tools used for quantitative studies (US National Heart, Lung, and Blood Institute quality assessment tool for observational, cohort, and cross-sectional studies [20], and quality assessment criteria proposed by Young and Solomon [21] and Davids and Roman [22]). Multimedia Appendices 1 and 2 present the 2 quality assessment tools used in this review. We assessed the intercoder consistency to determine the inclusion of articles for the review. All articles met the quality rating except for 1 article (the rating was <0.7 after the agreement of both researchers), which we thus removed from the final analysis.

The remaining 18 articles were then manually coded by 2 researchers based on preidentified themes: (1) patients’ discussion of online information during consultation, and (2) implications for the patient-physician relationship. During the coding process, both researchers independently identified subthemes and then added them to the existing themes upon agreement. Any disagreements were discussed and resolved before the final subthemes were confirmed. The first category included themes related to characteristics of doctor-patient consultations that led patients to reveal their online findings during visits with their doctor, such as strategies for using and revealing online information, facilitators of and barriers to discussion of online findings during consultations, and demographic factors affecting the discussion of online information. The second category, implications for the patient-physician relationship, focused on the influence of the patients’ Internet research on their relationship with the doctor, and include subthemes such as patients’ perception of a positive or negative impact on the patient-physician relationship, patients’ sense of control, confidence, and empowerment during the consultation, patients’ perceived consultation effectiveness, and patient satisfaction.

## Results

### Characteristics of Included Articles

Our initial PubMed and journal search returned over 4000 titles and citations. By applying the inclusion and exclusion criteria, we identified 744 records for further screening based on the title and abstract. Of these, we retained 316 articles for content screening, and then selected 19 peer reviewed journal articles that met the review criteria. Of the rejected articles, 228 focused on patients’ Internet health information seeking but did not address patient-physician relationship aspects. In 30 articles, the Internet was not the primary source of information. Of the remaining articles, 16 were not empirical studies, 11 were not about active information seeking, 5 were not available in full-text version, 3 were not in English, 2 focused only on postconsultation information seeking, and 2 focused on professional information seeking. Finally, we excluded 1 article among the 19 during the quality assessment procedure, leaving only 18 articles for the review. Of these 18 articles, 7 used surveys to gather data, 6 used interviews, 3 used semistructured interviews, and 2 used a mixed-methods approach. All articles were published within the period of 2003 to 2015 (see Table 1 and Table 2 for the complete list of articles and summaries) [3,4,7,12,15,23-35].

Of the 18 articles, 6 focused primarily on the implications for the patient-physician relationship, 2 studied the discussion of online information with physicians, and the rest studied both themes.

**Table 1:** Summary of articles on Internet-informed patients’ relationship with their physicians.

Study	Method	Country	Participant characteristics	Number of participants
Stevenson, 2007 [3]	Focus group interviews	UK	Adult patients with diabetes mellitus, ischemic heart disease or hepatitis C	34 patients (12 female, 22 male)
Kivits, 2006 [4]	Email interviews	UK	Users of UK websites devoted to healthy eating, fitness, and general health	31 (28 female, 3 male)
Broom, 2005 [7]	Interviews	Australia	Prostate cancer patients	33 male
Sommerhalder, 2009 [12]	Semistructured interviews	Switzerland	Patients and physicians from primary care and medical specialist practices	32 patients (12 female, 20 male) and 20 physicians (4 female, 16 male)
Murray, 2003 [15]	Telephone survey	US	Residents aged ≥18 years	3209 (1757 female, 1452 male)
Sillence, 2007 [23]	Observation and interviews	UK	Women faced with decisions concerning the menopause and hormone replacement therapy	15 female
Chung, 2013 [24]	Survey	US	Participants in the Health Information National Trends Survey (HINTS) 2007	5078 (3141 female, 1934 male)
Silver, 2015 [25]	Semistructured interviews	Canada	Community dwelling, ≥50 year, fluent in English, resident in Toronto, regular user of online health information	56 (30 female, 16 male)
Hart, 2004 [26]	Interviews	UK	Patients who had contacted health services in relation to hormone replacement therapy or menopause and Viagra or erectile dysfunction	47 (32 female, 15 male)
Schrank, 2010 [27]	Semistructured interviews	Vienna	Patients with schizophrenia or schizoaffective disorder	26 (12 female, 14 male)
Hay, 2008 [28]	Interviews and surveys	US	Rheumatology patients	120 (92 female, 28 male)
Newnham, 2006 [29]	Survey	Australia	Oncology patients just diagnosed with cancer	109 (44 female, 49 male)
Chiu, 2011 [30]	Focus group interviews	Taiwan	Cancer patients	46 (28 female, 18 male)
Russ, 2011 [31]	Survey	Israel	Patients at 10 primary care clinics	138 (82 female, 53 male)
Ybarra, 2008 [32]	Telephone survey	US	Participants in the national survey Surveying the Digital Future, Year 4	2010 (1214 female, 796 male)
AlGhamdi, 2012 [33]	Survey	Saudi Arabia	Patients at an outpatient clinic	801 (398 female, 400 male, 3 missing data)
Bianco, 2013 [34]	Survey	Italy	Adults aged ≥18 years	1039 (704 female, 335 male)
Xie, 2009 [35]	Interviews	US	Older adults, aged ≥60 years	20 (11 female, 9 male)

**Table 2:** Summary of findings of the articles.

Study	Themes	
	Discussion of online information with physicians	Implications for the patient-physician relationship
Stevenson, 2007 [3]	*Barriers to discussion of online information*: Patients experienced resistance from doctors over bringing information, even about their day-to-day health management, into the consultation. *Facilitators of discussion of online information*: Some patients reported that doctors positively encouraged them to search for information on the Internet. They also felt that Internet information should be checked with physicians.	*Quality of the patient-physician relationship*: Patients saw the Internet as an additional resource to support doctors’ advice and enhance the relationship with their doctor.
Kivits, 2006 [4]	*Strategies for using and revealing online information*: During consultations, patients preferred to be silent, asking questions and discussing information based on their Internet search, but not revealing that they used the Internet.	*Patients’ sense of control, confidence, and empowerment*: By discussing information they accessed on the Internet or setting questions in advance, patients mentioned being able to better understand and participate in consultation sessions with their doctors. Patients were also better informed, as they used the knowledge gained from Internet searches to check and complete the information received from doctors. Although most patients felt that physicians would feel challenged if the patients discussed information they found on the Internet, those who discussed the information said they had received positive attention.
Broom, 2005 [7]	*Barriers to discussion of online information*: Patients might feel being disapproved of by the physician if they shared their Internet search. Some physicians discouraged patients asking questions from their Internet research, giving them the impression that they were disapproved of or being treated as problematic patients.	*Patients’ sense of control, confidence, and empowerment*: Internet search provided clarity in terms of treatments options and, as a result, diminished patients’ reliance on their specialists. Further, Internet search behavior led patients to experience a heightened sense of control and therefore enter into a comprehensive negotiation with their specialist. However, patients’ sense of empowerment depended on how receptive providers and specialists were to their desire to take part in the decision-making process.
Sommerhalder, 2009 [12]	*Strategies for using and revealing online information*: Patients used several different strategies to introduce information found on the Internet to their physicians: ask additional questions; suggest specific diagnoses, diagnostics, or treatments, without directly revealing where they found the information; bring printouts of Internet search results into consultations; silently verify doctor’s advice against their online findings; actively avoid talking about the online information findings. *Barriers to discussion of online information*: Patients did not discuss their online findings due to lack of time during consultations, or reluctance to interfere with the consultation process.	*Patients’ perceived consultation effectiveness*: Patients viewed the consultation as important to their understanding of online health information. Physicians recognized the change in their role. Patients were more knowledgeable, which made initiating an interaction on health-related issues easier and enabled discussion on a more elaborate level; discussing with physician gave patients greater clarity, orientation, and certainty. *Quality of the patient-physician relationship*: Bringing up online information during consultations also resulted in conflicts with patients, and some patients ignored their physicians’ expertise.
Murray, 2003 [15]	*Demographic factors*: Those who brought information to the consultation tended to have a higher self-rated ability to critically appraise health information on the Internet and their health status.	*Patients’ sense of control, confidence, and empowerment*: Most felt more in control and more confident during the consultation as a result of bringing information to their physician. *Quality of the patient-physician relationship*: The effect of taking information to the physician on the patient-physician relationship was likely to be positive as long as the physician had adequate communication skills and did not appear challenged by the patient bringing in information. Patients’ who felt their physicians were challenged tended to be uninsured patients, and those who described themselves as excellent or very good at critically appraising information on the Internet.
Sillence, 2007 [23]	Not available.	*Patients’ perceived consultation effectiveness*: Patients felt that using the Internet improved their communication with physicians *Patients’ sense of control, confidence, and empowerment*: Patients felt better equipped to go to the physician and more empowered. The online information and advice influenced patients’ decision making without threatening their desire to communicate with physicians, but they still saw the physician as the primary source of information and advice.
Chung, 2013 [24]	*Demographic factors*: Men were more likely than women to have a conversation regarding online information with physicians. Patients who had trouble understanding or trusting online health information were no more likely to ask questions or seek guidance during consultations. Reactions of physicians to online information were perceived as negative by patients who experienced poor health and those who had more concerns about the quality of their searched information.	Not available.
Silver, 2015 [25]	*Barriers to discussion of online information*: Patients had not discussed or revealed their online health information findings due to fear of embarrassment; feeling it would be insulting to the physician; using online information to negate the need to see a physician; not remembering to bring it up. *Facilitators to discussion of online information*: Patients discussed online findings during doctor visits when a family member was present; the doctor initiated inquiries about patient-acquired information; they had encountered an advertisement suggesting talking with a doctor.	Not available.
Hart, 2004 [26]	*Strategies patients used to exchange online information*: Patients who looked up health information on the Internet prior to their consultation usually had not directly revealed to the practitioner that they had done so. *Barriers to discussion of online information*: Some practitioners sought to assert their authority by dismissing the discussion of patients’ findings acquired from the Internet.	*Quality of the patient-physician relationship*: Patients’ trust in their physician as the main information source remained at a very high level, despite their Internet health information searches.
Schrank, 2010 [27]	*Barriers to discussion of online information*: Patients feared their doctors could feel criticized if they revealed online findings or had an unchangeable preconceived view.	*Quality of the patient-physician relationship*: Online information showed the potential to significantly change the relationship with the attending doctors, with the most important aspect being a shift of the subjectively perceived hierarchy. The quality of existing patient-physician relationships played a major role in how patients assessed doctors when discussing online findings, where reactions were mostly judged as positive in a good relationship.
Hay, 2008 [28]	*Barriers to discussion of online information*: Patients did not discuss their Internet information seeking mostly because they feared being perceived as challenging or confronting their physician.	*Patient satisfaction*: Physician and patient appointment satisfaction was significantly higher when the Internet information was discussed.
Newnham, 2006 [29]	Not available.	*Quality of the patient-physician relationship*: Most patients did not believe that information searching adversely affected the doctor-patient relationship. 40% felt that the doctor-patient relationship was unaffected by information searching, 24% felt it improved the relationship, while only 8% felt it had adversely affected the relationship. 42% of patients who searched for information trusted their doctor as much as nonsearchers did.
Chiu, 2011 [30]	*Barriers to discussion of online information*: Patients worried that it might offend the doctors, they respected doctors’ authority, and were not used to asking doctors questions. *Demographic factors*: In a culture where the patient-physician hierarchy is prominent, patients were hesitant to ask questions, as it might displease the doctor.	*Patient perceived consultation efficiency*: Participants who searched the Internet before seeing their doctors could understand their doctors and the jargon they used better, thus leading to better doctor-patient communication.
Russ, 2011 [31]	*Demographic factors*: Those who presented information to their doctors tended to be older (average 43 years) than nonsharers (36 years, not significant), and information sharers tended to have more children under the age of 18 years.	*Patient satisfaction*: Patients who searched the Internet for information tended to feel that they received satisfactory information about their health during their consultation more than those who did not, and that they received more attention than the nonsharers.
Ybarra, 2008 [32]	Not available.	*Patients’ sense of control, confidence, and empowerment*: Most respondents felt more comfortable with information from the health provider as a result of their Internet searches.
AlGhamdi, 2012 [33]	Not available.	*Quality of the patient-physician relationship*: Of 801 study participants, 45% had searched for online health information before coming to the clinic; 72.5% of those discussed the information with their doctors, and 71.7% of those who did so believed that this positively affected their relationship with their doctor.
Bianco, 2013 [34]	Not available.	*Quality of the patient-physician relationship*: Only 25% of those who searched the Internet for health-related information discussed the information they found with their general physician. Most believed it had no effect on the patient-physician relationship, 13.4% believed the Internet information search had a positive effect, and only 8.1% believed it had a negative effect.
Xie, 2009 [35]	Not available.	*Patients’ sense of control, confidence, and empowerment*: A total of 4 online health information needs of patients were highlighted, of which 2 focused on the interaction with the physician: (1) advanced knowledge found on the Internet, on a specific health condition or treatment, helped patients to feel that they were better prepared to interact with doctors in the sense that they could better understand what doctors said; (2) the basic information about a health condition found on the Internet provided a general understanding of their health issue, so that it would help patients to know what to expect and to be prepared to better cope with a stressful situation.

### Discussion of Online Information With Physicians

Of the 18 articles reviewed, 12 examined patients’ discussion of information they found on the Internet with their physicians. These studies examined this category along 4 themes: (1) strategies patients use to reveal their Internet information searches, (2) facilitators of and (3) barriers to the discussion of online findings, and (4) demographic factors affecting discussion of online findings. Table 3 summarizes the themes and subthemes related to patients’ discussion of online information with physicians covered by each study. are summarized in Table 3.

**Table 3:** Themes and subthemes on patients’ discussion of online information with physicians.

Themes	Subthemes	Study reference												
		[3]	[4]	[7]	[12]	[15]	[24]	[25]	[26]	[27]	[28]	[29]	[30]	[31]
**Facilitators to discussion of online findings during consultations**														
	Having a family member present at physician visits							✔						
	Physician-initiated inquiries and encouraging patients to discuss	✔						✔				✔		
	Encountering a treatment-related advertisement that suggested talking with a physician							✔						
**Barriers to discussion of online findings during consultations**														
	Preestablished view of the patient-physician relationship							✔		✔	✔		✔	
	Physician resistance	✔		✔					✔					
	Perceived embarrassment							✔						
**Demographic characteristics**														
	Culture												✔	
	Sex						✔							
	Health literacy						✔							
	eHealth literacy					✔								
	Health status					✔								
	Age													✔
	Number of children <18													✔
**Strategies for using or revealing online findings during consultations**														
	Ask additional questions				✔									
	Make suggestions based on their online findings				✔									
	Directly disclose online findings				✔									
	Silently verify without asking any questions		✔		✔				✔					
	Bring printouts of online information				✔									

#### Strategies for Using and Revealing Online Information

A total of 3 articles examined strategies patients used to reveal their online findings during their doctor visits. These studies found 5 different strategies to be used by patients who brought online information to their consultations. These strategies were asking additional questions [4,12], making suggestions based on their online findings [12], directly disclosing online findings [12], verifying silently without asking any questions [4,12], and bringing printouts of online information [12]. Asking additional questions would allow patients to clarify contradictory points between their own view and the information from the physician. Making suggestions on different diagnostics and treatments would be helpful to patients in verifying their personal interpretations of online health information. Patients who preferred concealing their Internet search discussed online information without directly revealing that they had found the information on the Internet [4,12,26]. However, some patients preferred more accurate verification of their online findings by showing printouts of their Internet research to prompt discussions during consultations [12]. In fact, patients who directly disclosed online findings preferred critical appraisals from physician and appreciated their physician’s evaluations. Patients who silently verified their Internet search results did so to avoid interrupting the diagnosis process [12].

#### Facilitators of and Barriers to Discussion of Online Findings During Consultations

Silver [25] highlighted 3 facilitating factors that encouraged patients to discuss online health information with their physicians: (1) having a family member present at doctor visits, (2) doctor-initiated inquiries, and (3) encountering a treatment-related advertisement that suggested talking with a doctor. Having a family member present would help patients remember what to ask and made the context more comfortable to share online findings. Online advertisements or recommendations about certain medications and treatment options that contained information believed relevant to their own health condition prompted some patients to initiate a conversation with their physicians [25]. Further, some patients reported incidences of doctors’ positively encouraging patients to search the Internet for information [3]. These factors spurred patients to communicate their Internet research findings during consultations. In a study by Newnham et al [29], more than half of the patients who searched for online information prior to consultations had discussed information obtained in their search with their physician and had found their physician to be willing to discuss this information.

A total of 8 studies examined barriers to patients’ willingness to discuss their online findings with their physicians during consultations. The most common reason found was that patients were usually skeptical of how physicians would react to the knowledge they acquired through the Internet: patients were afraid doctors would perceive them as challenging doctors’ opinion if they directly revealed their online findings to their doctors [28]. Patients were mindful in ensuring that doctors played the central role during consultations [27]. They feared that revealing their knowledge gained from Internet searches would be an insult to professional health care providers [25] who could feel criticized or have an unchangeable preconceived view [27]. For example, Chiu [30] showed that patients cautiously made an effort not to offend doctors with their online findings. Patients expressed concerns over how physicians may perceive them as being “challenging” and “confrontational” if they discussed their health condition from a more informed point of view during consultations [28].

The second most common barrier for patients was the resistance or discouragement from physicians encountered when patients tried to discuss their Internet information research during consultations. Patients felt physicians’ resistance toward them when they tried to discuss with their physicians the health information they had found on the Internet on their conditions or even about day-to-day health management [3,7]. Patients also felt that some physicians reacted in a way that implicitly or explicitly discredited the patients’ ability to become informed via the Internet, presenting serious barriers to shared decision making during consultations, with the physicians asserting their authority by dismissing patient-acquired knowledge [7,26]. Patients felt that physicians were employing strategies to avoid online information-related dialogues or that they briefly answered patients’ queries with short answers to reclaim the traditional consultation model of one-way information provision. As a result, patients carefully observed their physicians before deciding whether to reveal their Internet research [25,30], and patients would only bring up their Internet health searches if they felt the situation was right.

A third major barrier was the fear of embarrassment [25]. Patients who identified this to be a barrier felt they did not possess the required skill set to evaluate online medical information. They had a lower level of confidence in the trustworthiness and the credibility of online information. They manifested a sense of being unsure of how to explain the information they found and how to relate it to their own condition, and hence did not want to mention it to their physicians.

Finally, other than the main barriers, some patients did not discuss their findings during consultations because they did not think the information was important enough and they searched the Internet just to be informed [15]. Other reasons for not revealing their online findings were a reluctance to interfere with physicians’ diagnostic process [12] and lack of time during doctor visits [12].

#### Demographic Factors Affecting Discussion of Online Information

The impact of patients’ demographic characteristics on their decision to discuss online health information with health care providers was studied in 4 studies. These studies examined demographic characteristics such as culture [30], sex [24], age, having children [31], health status [15], health literacy [24], and eHealth literacy [15]. Chiu [30] addressed the cultural influence on patient-physician encounters and patients’ Internet research. In a culture where the hierarchy of the patient-physician relationship is deemed to be like that of a son to a father, physicians have absolute authority to decide on the treatment, and patients must absolutely trust their doctors [36]. For such patients, even though online information empowered them with the knowledge to have a better discussion with doctors, they tended to do so cautiously, with an effort not to offend doctors and to assume greater responsibility in trying to understand their doctors’ advice with their knowledge gained from online health information.

The impact of sex was studied in a study by Chung [24], which showed that men were more likely than women to have a conversation regarding online health information with their physicians. Russ et al [31] showed that the average age of those who shared online information with doctors tended to be higher and they tended to have more children under the age of 18 years. Murray et al [15] found that people in poor health were more likely to talk to their physicians about online health information than were those in good health. Further, Chung [24] also showed that patients with low health literacy or who had trouble trusting online health information were not more likely to ask questions or to seek guidance during consultations. In contrast, Murray et al [15] showed that self-rated ability to critically appraise online health information was positively related to patients’ decision to discuss online information during consultations. Patients who rated themselves as excellent or very good at assessing the reliability of information on the Internet were more likely to take information to their physicians than were those who were not confident in assessing the reliability of Internet information [15].

### Implications for the Patient-Physician Relationship

A total of 15 articles studied the implications of patients’ online health information seeking for the patient-physician relationship. Of these, 8 studies focused on the patients’ perceptions of positive and negative implications for the patient-physician relationship, while 10 studies examined the indirect effects on the patient-physician relationship (ie, patients’ sense of control, confidence, and empowerment, perceived consultation efficacy, and patient satisfaction). Table 4 summarizes the themes and subthemes related to implications for the patient-physician relationship covered by each study.

**Table 4:** Themes and subthemes on implications of patient-physician consultation for the patient-physician relationship.

Themes	Subthemes	Study reference														
		[3]	[4]	[7]	[12]	[15]	[23]	[24]	[27]	[28]	[30]	[31]	[32]	[33]	[34]	[35]
**Patients’ perception of impact on patient-physician relationship**																
	Opportunity to discuss online findings	✔				✔						✔		✔	✔	
	Physician’s receptiveness to online information			✔	✔	✔										
	Prior relationship with patient								✔							
	Physician’s communication skills					✔										
	Patient demographics					✔		✔								
**Patients’ sense of control, confidence, and empowerment**																
	More in control and confident during consultation		✔			✔							✔			
	Heightened sense of empowerment			✔			✔									✔
**Patients’ perceived consultation effectiveness**																
	Better understanding of the illness condition		✔													
	Feeling better equipped during consultations to understand doctor						✔				✔					✔
	Greater participation in consultations		✔													
	More comfortable with doctor’s advice												✔			
	Provision of greater clarity, orientation, and certainty				✔											
**Patient satisfaction**																
	Satisfaction with the doctor’s advice											✔				
	Satisfaction with the appointment									✔						

#### Patients’ Perception of Positive or Negative Impact on the Patient-Physician Relationship

Of the 18 studies, 8 examined the factors directly affecting the patient-physician relationship. In the studies we reviewed, a greater proportion of participants were found to believe that Internet health information seeking did not adversely affect their relationship with physicians [3,29,33,34]. In the study by Newnham et al [29], 40% of patients felt the patient-physician relationship was unaffected by information searching, 24% felt it improved the relationship, and only 8% felt it adversely affected the relationship. However, the articles we reviewed showed that the effect of online information on the patient-physician relationship depended on several factors.

First, 5 studies showed the effect of patients’ discussion of their online findings with physicians. AlGhamdi and Moussa [33] reported that 45% had searched the Internet for health information before coming to the clinic; 72.5% of those discussed the information with their doctors, and 71.7% of those who did so believed that this positively affected their relationship. Patients who perceived their information search to have improved their relationship with physicians saw the Internet as an additional resource that supported doctors’ advice and enhanced the relationship with doctors [3]. They valued their relationship with their doctors and expected doctors to be more welcoming toward their Internet health research [15]. The positive influence of online information was stronger when patients had an opportunity to discuss their online findings [31,33,34].

On the other hand, bringing up online information during consultations also resulted in conflicts between patients and physicians. Conflicts stemming from different interpretation of online health information led to intensive discussions with physicians and patients [12]. Further, when patients valued the information they found on the Internet above their physicians’, this information led patients to ignore physicians’ expertise [12].

Second, Murray et al [15] found that how physicians reacted to patients when they shared their online findings during consultations could determine the positive or negative effect on the relationship’s quality. When patients perceived physicians to be threatened by their bringing online information, 49% of the patients were seriously dissatisfied with the consultation and 4% believed their relationship was worsened [15]. Bringing information was found to have a positive effect when the physician did not appear challenged by the online information [7,12,15].

Third, 1 study we reviewed showed the effect of physicians’ communication skills when patients discussed their online findings. Patients felt that the relationship was strengthened when physicians displayed adequate communication skills in discussing patients’ queries [15].

Fourth, Schrank et al [27] showed the influence of the quality of the existing relationship with physicians when patients assessed their physicians’ reaction during the discussion of online information. Patients judged their physicians’ reactions as mostly positive when they had a good prior relationship, even when the doctors’ replies were evasive or openly critical of the patients’ Internet search [27].

Fifth, 2 studies examined the influence of patients’ demographic characteristics on their assessment of physicians’ reaction to online information [15,24]. Murray et al [15] showed that most patients felt their physicians reacted positively to online health information, but those who felt their physicians were challenged tended to be uninsured patients, who described themselves as excellent or very good at critically appraising information on the Internet. Further, Chung [24] showed that physicians’ reactions to online information was perceived as negative by patients who experienced poor health, and they also had more concerns about the quality of the health information they sought on the Internet.

#### Patients’ Sense of Control, Confidence, and Empowerment During Consultation

A total of 5 articles reviewed examined the effect of Internet health information search on patients’ empowerment, perceived confidence, and control during a consultation. Murray et al [15] showed that patients felt more in control and confident during the consultation as a result of bringing information to their physicians. Patients also felt more confident in their physicians’ diagnosis once they had discussed their online findings [4,15,32]. Further, Internet search behavior led patients to experience a sense of control and therefore enter into a comprehensive negotiation with their specialist [7].

Of the studies we reviewed, 3 found that online health information can empower patients [23,35] to play a more active role in their disease management. A study of prostate cancer patients showed how the Internet affected their decision-making ability. Online information empowered them “to do something” rather than “just being told what to do” by their specialist [7]. Internet search provided clarity in terms of treatment options and, as a result, diminished patients’ reliance on their specialists.

Although Internet information search was shown to shift the subjectively perceived hierarchy between the doctor and the patient [27], patients still valued traditional doctor-patient consultations as important to their understanding of online health information [27]. The patients’ sense of empowerment was dependent on how receptive providers and specialists were to the patients’ desire to take part in the decision-making process [7]. Doctors’ resistance toward discussing online findings was found to result in higher levels of anxiety, confusion, and frustration.

#### Patients’ Perceived Consultation Effectiveness

In the studies we reviewed, most patients felt that Internet health information seeking prior to consultations had improved their communication with doctors and the effectiveness of their consultations. First, participants who searched for online health information prior to their consultations felt better equipped to communicate with their physicians during the consultations [23,35]. They believed the patient-physician communication had improved because they could understand their doctors and the jargon they used better [30]. Kivits [4] also found that, by discussing information they had accessed on the Internet or setting questions in advance, patients were able to better understand and participate in consultation sessions with their doctors.

Second, patients who searched the Internet for information prior to the consultation felt more confident and comfortable with the doctor’s advice. Ybarra and Suman [32] showed that a majority of patients had felt more comfortable with information from health care providers because of their Internet searches. Patients felt more informed as they used the knowledge gained from Internet searches to check and complete the information received from doctors. Further, discussions with physicians were found to give patients greater clarity, orientation, and certainty [12]. On the other hand, when physicians exerted resistance to patients’ online information sharing during consultations, it created a barrier to receiving effective care from the physician [7].

#### Patient Satisfaction

Only 2 studies examined the influence of patient satisfaction on the patient-physician relationship. Russ et al [31] found that online information seekers felt they had received satisfactory information about their health from their physician when compared with nonseekers. The appointment satisfaction of physicians and patients was found to be significantly higher when online health information was discussed [28], even if the information was not explicitly stated to be from the Internet. Patients who shared online information felt that they received more attention from their physician, compared with nonsharers [31].

## Discussion

### Principal Findings

Based on our review of the 18 empirical studies that examined patients’ Internet health information seeking and the implications for the patient-physician relationship, we found that a greater proportion of patients did not feel that their Internet health information-seeking activities had an adverse impact on the patient-physician relationship [3,29,33,34]. The recent proliferation of health information on the Internet has resulted in a shift in the traditional information balance [37,38], where patients are increasingly equipped with health information related to their conditions, eroding the prior exclusivity of health information among health professionals. However, our findings show that patients’ positive attitude toward physicians did not change unless physicians imposed restrictions on their online information sharing during consultations (eg, [3,7,26]). Patients went on the Internet mostly to be actively involved in the decision making related to their health. Patients still valued consultations with physicians [27], and their trust in physicians remained very high [26,27]. Patients used the information found on the Internet to help them prepare for their visit, ask better questions, and understand what the physicians told them. These were shown to empower patients to play a more active role in their disease management and to be more effective in understanding and communicating with their physicians [32]. Internet-informed patients were also more confident in and comfortable with their physicians’ advice [15].

In the studies we reviewed, some looked at how Internet health information seeking affected the patient-physician relationship, while others focused on how patients’ use of the online health information affected the patient-physician relationship. Although we identified 5 different types of strategies in the literature (including silently verifying information, bringing printouts, explicitly verifying information by asking questions, and asking extra questions without directly revealing their Internet search), most studies focused simply on whether patients discussed the online health information during physician consultations and the associated outcomes. Among these studies, evidence showed that patients experienced a better patient-physician relationship when they had the opportunity to discuss their online health information with their physicians, and their physicians were receptive to discussing the online information. However, if patients experienced resistance from their physicians to their discussion of online information, patients were found to become frustrated and anxious [7] and would withhold their discussion [3,7]. Conflicts arising from physicians and patients having different interpretations of the online information and when patients valued this information more also had adverse implications for the patient-physician relationship [12]. In general, we found more evidence of positive than of negative implications of discussing online health information.

As patients become better informed and like to be more actively involved in decision making about their health, traditional models of the patient-physician relationship need to be adapted to patients’ changing needs by incorporating their perspective into a relationship-centered medical paradigm [39]. In contrast to the physician-centric paternalistic models of care, a deliberative or participatory model has been recommended for encounters with Internet-informed patients [40], where physicians delineate the patients’ clinical situation and provide help in explaining and deciding on the available options [41]. Under this model of care, the physician acts as a teacher or a friend by engaging patients in a dialogue through the decision-making process [39].

Allowing or encouraging patients to discuss their Internet information searches with physicians is increasingly important, given that acquiring information on the Internet has the potential to misguide patients with inaccurate information and make them excessively anxious [8]. Therefore, the information patients wish to use in decision making ought to be verified to ensure that it is based on facts [40]. Additionally, not disclosing their Internet information searches could erode patients’ trust in their physicians if the diagnosis or the recommendations are different from their Internet research findings [2]. Our findings showed that enabling patients to communicate their Internet research was one of the key mechanisms to ensure that patients’ opinion was valued and to enhance physicians’ relationships with their Internet-informed patients. When physicians embrace openness to online information [7,12,15,24] and encourage patients to discuss the online information they have, patients’ perception of physician resistance and fear of embarrassment could be reduced and patients are more likely to discuss online information with their physicians.

### Research Gaps

In interpreting our findings, we should take note of the various research gaps in the existing studies. First, these empirical studies were primarily based on cross-sectional surveys, focus groups, or interview data, or a combination of these. Most of the results are descriptive, making it difficult to ascertain the causal effect of Internet health information seeking on the patient-physician relationship. In order to quantify the causal relationship between influencing factors and the quality of the patient-physician relationship, future research could involve more quantitative approaches, such as field experiments or surveys carried out in multiple waves. Second, the studies we reviewed focused mainly on understanding the patients’ perspectives, and hence our conclusions are limited to their perspectives, which might differ from those of physicians. Future research should explore physicians’ perspectives on patients’ Internet health information-seeking behavior and how physicians’ communication strategies during consultations could affect the patient-physician relationship.

### Limitations

We should also interpret our findings in the light of these limitations. First, the search criterion we used for retrieving the studies was initially broad to cover all the aspects that have been studied in relation to patients’ active Internet health information seeking. As there is no consistent terminology for the patient-physician relationship and its related dimensions, our main search query did not include MeSH terms. This may have resulted in missing out potentially relevant articles. However, we mitigated this limitation by performing a second round of search with a basic MeSH query. Second, we considered only articles that were in English. Therefore, we excluded several non-English articles from our review.

### Conclusion

Results of this review contribute to the understanding of the influence of health information sought by patients on the Internet on the patient-physician relationship. In contrast to the belief that patients’ Internet research can erode the patient-physician relationship [2], our findings show that patients’ Internet health information seeking has the potential to improve the relationship [3,27,29,33,34]. Patients typically see the Internet as an additional resource that can help them to better understand doctors’ recommendations and advice [3]. Thus, it has the potential to change the structure of the traditional patient-physician relationship [27,38] from one where patients perceive health care providers as the sole custodians of medical information [42]. Further research needs to be carried out to understand the needs and wants of Internet-informed patients, how physicians can adapt to this shift, and how traditional patient-physician relationship models must be adapted to meet the changing health paradigm.

## Appendix

Multimedia Appendix 1 Critical Appraisal Skills Programme (CASP) quality assessment for qualitative studies.

Multimedia Appendix 2 Quality assessment tool for quantitative studies.

## Abbreviations

MeSH: Medical Subject Headings
