# Supplementary material for: Internet Health Information Seeking and the Patient-Physician Relationship: A Systematic Review
Source: J Med Internet Res. 2017 Jan 19;19(1):e9. doi: 10.2196/jmir.5729 (PMC5290294; doi:10.2196/jmir.5729)
Supplement: Multimedia Appendix 1 [file jmir_v19i1e9_app1.pdf]

Multimedia Appendix 1: CASP (Critical Appraisal Skills Program) quality assessment for qualitative studies[a]

No	Question	Stevenson et al.[3]	Kivits [4]	Broom [7]	[12]Sommerhalder et al.	Sillence, et al. [23]	Silver [25]	Hart et al. [26]	Schrank, et al. [27]	Chiu [30]	Xie [35]
1	Was there a clear statement of the aims of the research?	1	1	1	1	1	1	1	1	1	1
2	Is a qualitative methodology appropriate?	1	1	1	1	1	1	1	1	1	1
3	Was the research design appropriate to address the aims of the research?	1	1	1	1	1	1	1	1	1	1
4	Was the recruitment strategy appropriate to the aims of the research?	1	1	1	1	1	1	0	1	1	1
5	Was the data collected in a way that addressed the research issue?	1	1	1	1	1	1	1	1	1	1
6	Has the relationship between researcher and participants been adequately considered?	0	0	0	0	0	0	0	0	0	0
7	Have ethical issues been taken into consideration?	1	0	1	1	0	0	1	1	0	0
8	Was the data analysis sufficiently rigorous?	1	1	1	1	1	1	1	1	1	1
9	Is there a clear statement of findings?	1	1	1	1	1	1	1	1	1	1
Outcomes of the research											
10i	Researcher(s) have discussed the contribution of the study to the existing knowledge or understanding:	1	1	1	1	1	1	1	1	1	1
10ii	Researcher(s) have identified new areas where research is necessary:	1	1	0	1	1	1	1	1	0	1
10iii	Paper has addressed whether or how the findings can be transferred to other populations or considered other ways the research may be used:	1	0	0	1	1	1	1	1	1	1
	**Quality score[b]**	0.92	0.75	0.75	0.92	0.83	0.83	0.83	0.92	0.75	0.83

a: The results presented in the table are after resolving the disagreements between two researchers

b: Yes = 1, No = 0 and Can’t tell = 0, the total score was calculated based on the proportion of ‘Yes’
