# Supplementary material for: Internet Health Information Seeking and the Patient-Physician Relationship: A Systematic Review
Source: J Med Internet Res. 2017 Jan 19;19(1):e9. doi: 10.2196/jmir.5729 (PMC5290294; doi:10.2196/jmir.5729)
Supplement: Multimedia Appendix 2 [file jmir_v19i1e9_app2.pdf]

Multimedia Appendix 2: Quality assessment tool for quantitative studies[a]

No	Questions	Murray, et al. [15]	Chung [24]	Hay, et al. [28]	Newnham, et al. [29]	Russ, et al. [31]	Ybarra and Suman [32]	3]AlGhamdi and Moussa	Bianco, et al. [34]
Objectives									
1	Was the research question or objective in this paper clearly stated?	1	1	1	1	1	1	1	1
2	Was the study population clearly specified and defined?	1	1	1	1	1	1	1	1
Sample selection and method									
3	Was the participation rate of eligible persons at least 50%?	1	2	1	1	1	2	1	1
4	Sampling method: Was it representative of the population intended in the study?	1	1	1	1	1	1	1	1
5	Was a sample size justification, power description, or variance and effect estimates provided?	1	1	0	0	1	0	0	1
6	Was the study design appropriate for the research question?	1	1	1	1	1	1	1	1
8	Was it a primary or secondary data source?[yes = primary, no = secondary]	1	0	1	1	1	0	1	1
9	Does the study test a stated hypothesis?	0	0	0	0	0	0	0	0
Design and Results									
10	Were the independent variables clearly defined, valid, reliable, and implemented consistently across all study participants?	1	1	1	2	1	1	1	1
11	Were the outcome measures (dependent variables) clearly defined, valid, reliable, and implemented consistently across all study participants?	1	1	1	2	1	1	1	1
12	Were the statistical analyses performed correctly?	1	1	1	1	1	1	1	1
13	Do the data justify the conclusions?	1	1	1	1	1	1	1	1
Outcomes of the research									
14i	Researcher(s) have discussed the contribution of the study to the existing knowledge or understanding:	1	1	1	1	1	1	1	1
14ii	Researcher(s) have identified new areas where research is necessary:	0	1	0	1	0	1	0	1

14iii	Paper has addressed whether or how the findings can be transferred to other populations or considered other ways the research may be used:	0	1	1	0	1	1	1	1
	**Quality score[b]**	0.80	0.86	0.80	0.77	0.87	0.79	0.80	0.93

a: The results presented in the table are after resolving the disagreements between two researchers
b: Yes = 1, No = 0, Not available = 0, and Not relevant = 2, the total score was calculated based on the proportion of ‘Yes’, after omitting ‘Not Relevant' questions
